# A randomized controlled study about the use of eHealth in the home health care of premature infants

**DOI:** 10.1186/1472-6947-13-22

**Published:** 2013-02-09

**Authors:** Anna Gund, Bengt Arne Sjöqvist, Helena Wigert, Elisabet Hentz, Kaj Lindecrantz, Kristina Bry

**Affiliations:** 1 Department of Signals and Systems, Chalmers University of Technology, 412 96, Gothenburg, Sweden; 2 Institute of Health and Care Sciences, The Sahlgrenska Academy at University of Gothenburg, 416 85, Gothenburg, Sweden; 3 Division of Neonatology, Sahlgrenska University Hospital, 416 85, Gothenburg, Sweden; 4 School of Technology and Health, KTH - Royal Institute of Technology, 141 52, Huddinge, Sweden; 5 Department of Clinical Science, Intervention, and Technology, Karolinska Institutet, 141 46, Stockholm, Sweden; 6 Department of Pediatrics, The Sahlgrenska Academy at University of Gothenburg, 416 85, Gothenburg, Sweden

**Keywords:** Video conferencing, Internet, Preterm infant, Home health care, eHealth, Web application

## Abstract

**Background:**

One area where the use of information and communication technology (ICT), or eHealth, could be developed is the home health care of premature infants. The aim of this randomized controlled study was to investigate whether the use of video conferencing or a web application improves parents’ satisfaction in taking care of a premature infant at home and decreases the need of home visits. In addition, nurses’ attitudes regarding the use of these tools were examined.

**Method:**

Thirty-four families were randomized to one of three groups before their premature infant was discharged from the hospital to home health care: a control group receiving standard home health care (13 families); a web group receiving home health care supplemented with the use of a web application (12 families); a video group with home health care supplemented with video conferencing using Skype (9 families). Families and nursing staff answered questionnaires about the usefulness of ICT. In addition, semi-structured interviews were conducted with 16 families.

**Results:**

All the parents in the web group found the web application easy to use. 83% of the families thought it was good to have access to their child’s data through the application. All the families in the video group found Skype easy to use and were satisfied with the video calls. 88% of the families thought that video calls were better than ordinary phone calls. 33% of the families in the web group and 75% of those in the video group thought the need for home visits was decreased by the web application or Skype. 50% of the families in the web group and 100% of those in the video group thought the web application or the video calls had helped them feel more confident in caring for their child. Most of the nurses were motivated to use ICT but some were reluctant and avoided using the web application and video conferencing.

**Conclusion:**

The families were satisfied with both the web application and video conferencing. The families readily embraced the use of ICT, whereas motivating some of the nurses to accept and use ICT was a major challenge.

## Background

eHealth, the use of information and communications technology (ICT) in health care, [1,2], is a steadily growing area of interest. Technological advances and increasing use of ICT among the general public have driven health care providers to develop new ICT tools and applications for improving the quality of care and services provided to the patients. Sweden has already adopted several eHealth services in practical health care. For example, all primary care units in Sweden today have access to electronic health records [3], and more than 80% of all prescriptions are now transferred electronically [4].

Neonatology, the care of the diseases of newborn infants, is a field where eHealth has not yet been applied to any significant extent, but where it could potentially be useful. Because of their immaturity and associated illnesses, premature infants (i.e. infants born at gestational ages 23–37 weeks) often need hospital care at a neonatal intensive care unit (NICU) for several weeks after birth. The illness and prolonged hospitalization of the infant are very stressful for the parents and inhibit the development of a normal parent-infant relationship [5]. Infants who have become sufficiently mature and physiologically stable can be taken care of in the home environment. This usually requires that the infant has reached a postmenstrual age of at least 34 weeks. Caring for the child at home has been shown to be beneficial for families, since the parents can connect with their child better than is possible in the hospital [5]. It also decreases the newborn’s exposure to bacteria prevalent in neonatal intensive care units [6,7]. In recent years, the Sahlgrenska University Hospital in the Gothenburg area in Sweden has had a home health care program for newborn infants. The medical issues that these children have include poor weight gain or need of tube feeding or supplemental oxygen therapy. During the first weeks at home the family is supported by regular visits 2–3 times per week by a neonatal nurse who weighs and measures the child, helps the parents with the care of the child, and answers questions that come up.

Having the baby at home can initially be difficult for parents who may not yet feel confident taking care of their infant and who often have concerns and questions about the infant’s health and development [8-10]. On the other hand, home visits by nurses represent a large investment of resources, since one nurse can only make a few visits per day.

Already in 1994, a project on using telemedicine/eHealth in the care of prematurely born infants in the home was performed in Gothenburg [11]. In this project a system for monitoring of infants with respiratory distress syndrome in need of oxygen therapy was developed and adapted to a home environment. The system was accepted by the participating families, and the project showed that telemedicine/eHealth applications are adaptable for use in care of infants at home.

Another uncontrolled study where video conferencing was offered to a small group of parents whose premature infants were discharged from the hospital showed that parents experienced video conferencing as positive [12]. The families only had support through the video conferencing system not complemented by visits by a nurse [12]. In the United Kingdom, two studies showed that video conferencing could be used to provide support for parents of children born with congenital heart disease after discharge from the hospital [13,14]. In this study, video conferencing, which was used to supervise the children visually as well as to communicate with the parents, was found to be more useful than ordinary phone calls [13,14]. In the Baby CareLink study, families of hospitalized very low birth weight infants could use a videoconferencing module for virtual visits from their home to the NICU and a website to access information on the issues confronting them [15]. This study showed that the use of videoconferencing and Internet support increased the parents’ satisfaction with the care of the infants and facilitated earlier discharge home of the infants [15]. Prototype systems have been developed for monitoring and tracking observations of behavioral and health-related data of premature infants in the home [16]. The FitBaby prototype includes a mobile phone-based application for parents entering observations about their infant’s health as well as a networked database in which these data are stored for clinician access [16,17]. Estrellita is a prototype system that includes a mobile application that parents use to track and share information and a web interface that parents and health professionals use to view and analyze the resulting data [18]. This system gives parents the ability to easily share information with social networks and health care professionals [18]. NICU-2-HOME is a project in the USA that deals with making the transition from hospital to home easier for the parents with prematurely born infants using a mobile application [19].

In the present study, we hypothesized that use of eHealth would improve communication between parents of premature infants and home health care nurses and enhance the families’ feeling of confidence in caring for their infant. The families were randomized to one of three study groups, one receiving standard home health care, one receiving home health care supplemented by the use of a customized web-based eHealth system, and a third group receiving home heath care supplemented by video conferencing using Skype to communicate with neonatal nurses. The results show that the telecommunication methods were well accepted by the parents and that their use made parents more confident taking care of their infant at home. Many families also felt that telecommunication could decrease the need of home visits and could therefore make home health care more cost-effective.

## Methods

### Approval by the institutional review board

The study was approved by the Regional Research Ethics Committee in Gothenburg.

### The web application

Care@Distance [20] is a generic web-based system solution through which healthcare professionals and patients can communicate and exchange health-related information using various webpages tailored to fit specific clinical applications and needs. All users can access the web application through any of the most commonly used web browsers, e.g. Microsoft Explorer. Consequently no application-specific software needs to be installed by either patient or caregiver. All information is stored in a Microsoft SQL Server 2005 database, on a virtual server running Microsoft Server 2008. The web application itself is written in a combination of PHP, HTML and Javascript.

**Figure 1:**
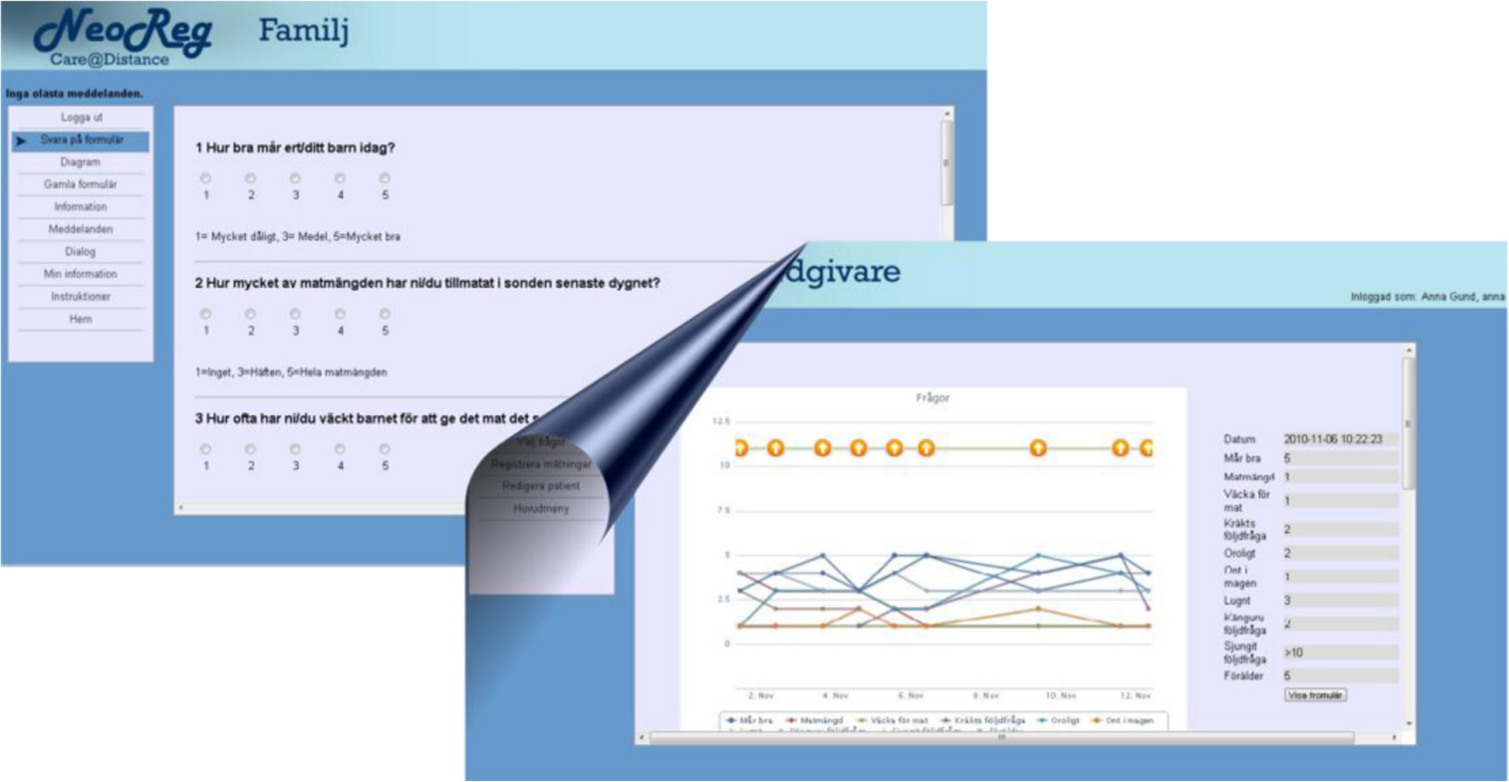
Screenshots of web pages from the web application used in the study.

The neonatal application of Care@Distance contains a formulary (Figure 1) with questions about the child’s health and nutrition status as well as about how the parents were coping with the care of the infant. The families could view the data that they or the health care staff had entered, including the child’s weight and head circumference data, in graphic form as well as exchange internal messages with the staff. The application also includes general information about preterm babies’ health, development, and medical conditions that the families could access. The nursing staff could view the data that they or the parents had entered, enter specifics about the patients’ health, administer the system (e.g. add new families, change passwords, edit forms, etc.), and exchange internal messages with both families and other staff.

### Internet calls using Skype

Skype is a commercially available, widely used, off-the-shelf program for video conferencing over the internet. It is free of charge, and easy to install and use. The user logs in with a username and a password and can call other Skype users with voice or video calls through a contact list, and also send them text messages. Hardware requirements are a Windows or Macintosh computer with internet access. For video calls, a web camera, a microphone, and speakers are necessary. For video calls, Skype recommends a broadband connection with a minimum of 128 kilobits per second down- and upload speed [21]. For high definition (HD) video calls the minimum requirements are 1.2 Megabits per second down- and upload speed [21].

### Eligibility of patients, randomization, and study groups

All families with preterm babies who were treated at Sahlgrenska University Hospital and who were going to be assigned to health care at home were eligible for the study if they did not meet any of the following exclusion criteria: the parents had inadequate knowledge of Swedish, the parents did not have a computer and internet connection at home, the infant was expected to need home health care for a very short time (less than a week), or the parents did not want to use a computer or a web camera.

After informed consent, the families were randomized into one of three groups. Group 1 was a control group receiving standard home health care. Group 2 (web group) had home health care complemented with the web application, and were asked to answer the questions presented there once a day. They were also encouraged to write individual messages or questions to the nurses either through the form or through the internal mail function. If interested, the families could follow their child’s progress using the web application graphs. In addition to oral explanation the families were given written instructions about the use of the web application. The families in group 3 (video group) had similar home care to those in Group 1, except that they had video conferencing with nurses instead of the traditional phone calls. The families in group 3 were given a bag containing a HD web camera (Logitech QuickCam Pro9000 for PC users and Logitech QuickCam Vision Pro for Mac users), a set of speakers (Ace G35) and a compact disc containing the installation program for Skype version 5.3. The families were given both oral and written instructions about the use of the web camera and Skype. Each family participated in the study for as long as they were included in the home health care program.

### Nursing personnel

Six nurses at the hospital were involved in the home health care program. All of them participated in the study. Each nurse worked in home health care for a period of 6 weeks and then for 12 weeks either at the level II nursery or at the level II of the hospital. At any given time, two of the nurses did home visits, two worked at the level II nursery, and two at the NICU.

When it was clear that a baby would eventually receive home health care, a nurse at the hospital gave the family information about the home health care and about the study, obtained informed consent, and randomized the family. The nurse who subsequently took care of the family at home was not necessarily the same one who had given the information and randomized the family. The family was allocated to a home health care nurse depending on the number of patients each nurse had and, to minimize driving times, on the area where the family lived. Although it was preferable to have only one nurse during the whole home health care period, this was not always possible due to the rotation and vacations of the nurses. All the nurses were trained in the use of both the web application and Skype.

All three patient groups received regular home visits where the nurse measured the weight and head circumference of the child. The nurse also helped the parents take care of the baby, assisted with tube and breast feeding, answered any questions the parents might have, and changed the nasogastric feeding tube when indicated.

For the families in the web group, the nurses were supposed to enter the weight and head circumference data of the children into the web application. They were also supposed to give daily feedback though the application to the families who had sent a report about their child’s health status and to answer any questions the parents had presented via the application.

In all groups, the nurses called the families to check on how the baby was doing and to make plans for the next home visit. The families in the Skype group were supposed to receive video calls instead of regular telephone calls. The equipment the nurses used was a laptop PC (Asus K72DR-TY030V) with HD web camera (Logitech QuickCam Pro9000) and speakers (Logitech X-140) or a headset (Logitech Headset Premium).

### Evaluation methods

#### Parent questionnaire

After discharge from the home health care program, the families were asked to answer a questionnaire about their experience of various aspects of the home health care. The questionnaire contained questions about the home health care program in general and about the home visits. These questions were the same for all the three groups. In addition, the Skype group and the web group had questions concerning their respective methods. Most questions were answerable on a five-point Likert-like scale, and for the rest, three alternatives were given (yes, no, or no opinion). Families were also encouraged to write freely worded comments.

#### Interviews of parents

Semi-structured interviews were performed on 16 families, 7 in the control group, 6 in the web group, and 3 in the Skype group. All parents were interviewed in their homes. The interviews lasted from 45 min to 1 hour and were audio-recorded and transcribed. The interviews were conducted mean 5.5 ± 0.84 months after the infant was discharged from home health care.

#### Nurse questionnaire

In order to get information about nurses’ experiences of and attitudes about the use of the web application or of Skype, they were likewise asked to fill out a questionnaire that contained questions on the usage and user acceptance of both the web application and communication by Skype.

### Presentation of numerical data

Data is expressed as means ± standard error of the mean (SEM) or as median (range). Gestational ages are expressed as weeks (wks) + days (d).

## Results

### Characteristics of the mothers and infants

Characteristics of the mothers and infants are shown in Table 1. The mean age of the mothers was 31 ± 1 years. The infants’ gestational ages varied from 25 wks + 6d to 37 wks + 4d. Most infants were moderately premature, since the median gestational age was 33 wks ± 5d. They were discharged to home health care when they were physiologically stable and did not have apnea, and when the family had received sufficient training to be able to take care of the infant at home. At that time, the mean postmenstrual age of the infants was 35 wk + 2 d. The mean length of stay in the home health care program was 20 ± 1 days. The mean postmenstrual age at which the infants were discharged from home health care was 38 wks + 1 d.

**Table 1:** Characteristics of the mothers and infants

	**Mean ± SEM**	**Median (range)**
**Mothers**		
Age (years)	30.9 ± 0.9	31 (23–40)
**Infants**		
Gestational age (weeks + days)	33 wks + 2 d ± 3 d	33 wks + 5 d (25 wks + 6 d– 37 wks + 4 d)
Birth weight (g)	2073 ± 105	1948 (870 – 3750)
Postmenstrual age at the time of discharge from hospital to home health care (weeks + days)	35 wks + 2 d ± 2 d	35 wks (35 wks + 2 d – 42 wks + 2 d)
Length of stay in home health care (days)	20 ± 1.4	18.5 (9–39)
Postmenstrual age at the time of discharge from home health care (weeks + days)	38 wks + 1 d ± 2 d	37 wks + 4 d (35 wks + 2 d– 42 wks + 2 d)

### Families’ opinions about the home health care in general

Almost all the parents were very satisfied with the home health care (Table 2). They were also well prepared for the transfer of the infant from the hospital to home health care, confident with the care of their infant at home, and satisfied with the home visits by the nurses (Table 2). All but two families (94%) felt that the number of home visits was appropriate, one family (3%) felt that they would have needed more visits, and one (3%) had no opinion. All the families thought that the visits were long enough and that the instructions given by the nurses during the home visits were easy to understand.

**Table 2:** Opinions of the parents about the home health care in general

**Questions with Likert-like scale**	** 1**	** 2**	** 3**	** 4**	** 5**
How satisfied were you with the home health care in general?	0 (0%)	0 (0%)	0 (0%)	3 (9%)	29 (91%)
How well prepared were you when your baby was discharged from the hospital to home health care?	0 (0%)	0 (0%)	0 (0%)	10 (31%)	22 (69%)
How confident did you feel in the care of your baby at home?	0 (0%)	0 (0%)	0 (0%)	7 (22%)	25 (78%)
How satisfied were you with the home visits by the nurses?	0 (0%)	0 (0%)	0 (0%)	2 (6%)	30 (94%)
**Questions with No/Yes answers**		**No**		**Yes**	
Were the home visits stressful for your family?		31 (97%)		1 (3%)	
Did you have a sufficient number of home visits?		1 (2%)		31 (97%)	
Were the home visits long enough?		0 (0%)		32 (100%)	

The number of families is shown with the percentage in parentheses. For the Likert-like scale, 1 = very dissatisfied, very poorly prepared, very insecure; 0 = neutral (neither/nor); 5 = very satisfied, very well prepared, very confident.

In their free-response comments on the questionnaires as well as during the semi-structured interviews, all the parents expressed great satisfaction with the home care they had received.

“It was luxurious to have someone come and meet us in our own home. Very nice and pleasant.”

They were relieved and happy to be able to leave the hospital and felt that the home visits helped them to feel confident at home with their baby.

“It was great that the nurse came, it was so nice to know that she would come in a day or two and one could ask her if one was wondering about something.”

All the parents reported that the visits at home worked very well. They felt that the nurses were very competent and friendly and appreciated the possibility of being able to discuss problems and ask questions without feeling time pressure. The parents particularly appreciated being able to contact the hospital neonatal unit at any time of the day, although few of them needed to call.

“Wonderful to have nursing staff within easy reach by phone if there was something and very convenient that they came home.”

### Results specific to the use of the web application

#### Computer experience of the families

All the families estimated that their computer experience was extensive (Table 3). All the parents used computers at home and 11 of 12 (92%) used computers also at work.

**Table 3:** Opinions of the families about using the web application

**Questions with Likert-like scale**	** 1**	** 2**	** 3**	** 4**	** 5**
How extensive is your computer experience?	0 (0%)	0 (0%)	0 (0%)	2 (16.7%)	10 (83.3%)
How good is your general impression of the web application?	0 (0%)	0 (0%)	2 (16.7%)	6 (58.3%)	4 (37.5%)
How easy was the web application to use?	0 (0%)	0 (0%)	0 (0%)	2 (16.7%)	10 (83.3%)
**Questions with Yes/No answers**	**No**		**Yes**		**No opinion**
Was it good to be able to view your child’s data?	1 (8.3%)		10 (83.3%)		1 (8.3%)
Did you get feedback from the nurses regarding the data you entered?	3 (25%)		9 (75%)		0 (0%)
Did you get answers to any questions by using the information link?	1 (8.3%)		11 (91.7%)		0 (0%)
Did the webpage help you feel more confident in the care of your child at home?	6 (50%)		6 (50%)		0 (0%)
Do you feel that the application reduced your need of home visits?	5 (66.7%)		3 (33.3%)		0 (0%)

The number of families is shown, with the percentage given in parentheses.

For the Likert-like scale, 1 **=** very slight, very bad, very difficult, much more stressful; 0 = neutral (neither/nor); 5 = very extensive, very good, very easy.

#### Web application: ease of use, content, and usefulness

The general impression of the web application was positive. Ten out of 12 families (83%) found the application to be good or very good, and the rest thought it was neither good nor bad. All the families found the application easy to use (Table 3).

Most families answered the questions about their baby’s health every day.

During the interviews, the families described their positive experiences as follows:

“The webpage was another possibility of contacting the home care nurse. It was easy to use, a complement to the home visits. Answering the questions on the webpage was an opportunity for reflection, how has the day been, how has our daughter been.”

“The questions on the webpage reminded us of what is important for our child.”

“The webpage gave us a possibility of more frequent updates than the home visits.”

On the other hand, some of the infants were so stable throughout the period they were included in home health care that the families felt no need for the web application:

“The webpage was not so interesting for us since she was so healthy and easy to take care of that it did not make that much difference.”

“We wrote the same answers every day since our daughter was doing so well. We could have been fine without the webpage, but it was nice to have it anyway. It was nice that the nurse knew how we were doing when she came so we did not need to explain it to her during the home visits.”

The answers entered by the parents and the weight and head circumference measurements of the infant entered by the nurses could be seen in graphic form utilizing the web application. Thereby the progress of the infant could be easily visualized. This feature was appreciated by the parents.

The nurses were supposed to read the parents’ answers and send a comment back to them. Most families (8 of 12 families, or 67%) reported having received feedback from the nurse about the data they entered both through the applications message function and during home visits. However, one family (8%) felt that it had received very little feedback and 4 families (25%) claimed they had received no feedback from the nurse about the information they had entered. Several families commented about the importance of getting feedback.

For acute questions, the families were asked to call the neonatal nursery at the hospital, but in less urgent cases, they could ask the home health nurse questions through the web application. The families reported having asked the nurses about the infant’s weight, nutrition, vomiting, stooling pattern, and stomach problems, as well as about the use of skin-to-skin. In most cases, the nurses answered the question the same day or the following morning. All of the families participating in the study had the possibility of reaching the nurses at any time by calling on their mobile phone. Some families commented that although using the web application was easy, calling the nurses on the phone was even more straightforward and that they preferred to have a personal contact with the nurse.

“The web was like a phone call with a long response time. Some phone calls could perhaps be replaced by messages, but it seems more convenient to call the nurse directly.”

“We could use the web instead of calling. But often when you wonder about something, you want an answer right away.”

“We had the possibility of calling the hospital in the evenings and at night. To call was more natural, more personal. We had some questions we wanted answered directly.”

“We could always call the hospital. The web is an anonymous way of communicating, a quiet anonymous communication. You want to speak to a real person. Communication with a person is really important to make one feel reassured.”

The web application included a link to an information booklet compiled by the Division of Neonatology so that the parents could find information about various aspects of the care of a newborn and of a premature infant. 92% of the families got information and answers to their questions using this link. Only one family had not looked for information in the booklet.

A third of the families would have wanted to add some features to the web application. Additional features desired by the families were a better diagram function, direct contact with the nurse through e.g. chat, compatibility with other web browsers, daily e-mail reminders to answer the questions, and more links to sites containing information about the growth and development of premature infants. One family would have liked to be able to contact other families with premature infants through the web application.

50% of the families felt that the web application had helped them feel more confident in the care of their child. 33% thought that its use could reduce the need of home visits, although not completely replace them.

### Results specific to the use of Skype

All the families found Skype easy to use and were satisfied with the video calls (Table 4). Seven out of the eight families (87.5%) thought it was easy to communicate with the nurse through video calls. One family, which had had only one video call, thought communicating through video calls was neither easy nor difficult. Likewise, 87.5% of the parents had received answers to their questions during the video calls and felt that the instructions they received during the Skype calls were important to them. 50% of the families felt that the video calls were less stressful than home visits (Table 4).

**Table 4:** Opinions of the families about using Skype

**Questions with Likert scale**	** 1**	** 2**	** 3**	** 4**	** 5**
How easy was it to use Skype?	0 (0%)	0 (0%)	0 (0%)	4 (50%)	4 (50%)
How satisfied were you with the video calls?	0 (0%)	0 (0%)	0 (0%)	5 (62.5%)	3 (37.5%)
How easy was it for you to communicate with the nurse by video call?	0 (0%)	0 (0%)	1 (12.5%)	2 (25%)	5 (62.5%)
Were the answers or instructions you received during the video calls useful to you?	0 (0%)	0 (0%)	2 (25%)	4 (50%)	2 (25%)
Were video calls less stressful than home visits?	0 (0%)	0 (0%)	4 (50%)	2 (25%)	2 (25%)
**Questions with No/Yes answers**		**No**		**Yes**	
Did you receive answers to your questions during the video calls?		1 (12.5%)		7 (87.5%)	
Were video calls better than ordinary phone calls?		1 (12.5%)		7 (87.5%)	
Did the video calls help you feel more confident in the care of your child at home?		0 (0%)		8 (100%)	
Do you feel that the video calls reduced your need of home visits?		2 (25%)		6 (75%)	

The number of families is shown, with the percentages given in parentheses.

For the Likert-like scale, 1 = very difficult, very dissatisfied, very useless, much more stressful; 0 = neutral (neither/nor); 5 = very easy, very satisfied, very useful, much less stressful.

Seven of the eight families (87.5%) considered video calls better than ordinary phone calls. The family that had received only one video call thought that a telephone call would have been as good. All the other families commented on the questionnaire that seeing the nurse improved communication. Although the possibility of showing the child to the nurse was appreciated, only two families had shown their child to the nurse using the web camera.

“It is easier to understand when you can see the other person’s body language.”

“A picture emphasizes the words.”

“It was possible to show the child to the nurse if there was a problem that could be “seen”.”

Importantly, all the families reported that the video calls helped them feel more confident in the care of their child at home (Table 4). Moreover, 6 of 8 families (75%) thought that video calls could reduce the need of home visits. One of these felt that the home visits could completely be replaced by video calls. During the interviews, the families belonging to the Skype group commented on replacing home visits with Skype calls as follows:

“I think Skype could have replaced every other or every third visit.”

“For me it would have worked well with Skype. It would have worked for me 80% of the time, but 20% of the visits are indispensable. That someone comes and looks at her.”

“I think Skype could have replaced some visits. It worked great.”

Overall, the Skype families’ comments on the questionnaires and during the interviews revealed their enthusiasm for the video calls:

“Skype is a great solution and we hope more parents will be able to use it.”

“A very interesting project that certainly can develop home health care.”

“A great form of health care – we are so pleased!”

“We sat with V. (the child) in our lap like a little family photo. It was great. It is wonderful that such progress exists, new possibilities for children.”

Families in the control group also commented during the interviews on the possibility of using video calls in home health care:

“It would be possible to combine Skype and home visits.”

“If we had had Skype, we would have seen one another and you could show the child’s equipment and get instructions on how to use it. It could have replaced visits where the nurse did not have to do anything, such as weigh the baby.”

“We hoped that we would have been assigned to the Skype group. It would have been a great complement to home visits. It would have felt safer to be connected to the hospital in this way.”

Some families in the web group also had opinions about the use of Skype:

“We think that Skype could have been better than the home page. We hoped we would be assigned to the Skype group. We are used to talking to our parents with Skype.”

“If we had had Skype, it would have been sufficient to have a home visit only every other week. To replace home visits, Skype would have been better than the webpage.”

### Opinions and attitudes of the nurses about the use of telecommunication in home health care

#### Results from the nursing personnel questionnaire

Five of the six home health care nurses answered the questionnaire, and one chose not to contribute. The five nurses who answered the questionnaire had a mean age of 48 ± 3 years, median age of 50 years (range 38 to 56), and were thus significantly older than the parents.

The youngest nurse estimated her computer experience as extensive, three nurses as moderate, and one as slight, although the hospital has used electronic patient charts for over a decade and thus all the nurses used the computer daily at work for writing nursing reports for patient charts. Four nurses (80%) used the internet socially daily, whereas one nurse (20%) seldom used the internet. Only one nurse (20%) used Skype privately.

All the nurses were allowed to inform and recruit families for this study as soon as it was clear that the family would eventually receive home health care. However, it turned out that two of the nurses informed a much larger number of families (17–18) than the other three (0–9). They also had a much higher acceptance rate (80-90%), compared to 30-45% for the others. The nurses who had the lowest acceptance rate also thought that the families were less interested in the study than those with high acceptance rate. Overall, 70% of the families accepted participation in the study.

The nurse informing the family was generally not the same one who subsequently would make home visits to the family. Due to the rotation and vacations of the nurses, the family could have more than one nurse during their stay in the home health care program. 59% of the families had the same nurse during the whole duration of the home health care, 35% had two different nurses, and 6% had three different nurses.

Most (80%) of the nurses thought the study was good or very good. The nurse with least computer experience thought the study was neither good nor bad. The freely worded comments of the nurses revealed differences in the attitudes of the nurses about the study:

“I think the study is interesting. I am interested in developing the care of the infants and in finding new possibilities. It is important that care keeps pace with the society’s technological development.”

“We can use these new communication methods in home health care.”

“I have not learned to use the methods well enough. It is mostly my own fault.”

#### Nurses’ opinions about the web application

All the five nurses who answered the questionnaire used the web application during the study. All of them thought the web application was good. Most of the nurses found it easy to use.

80% of the nurses thought that the parents appreciated the application, and 60% thought that it contributed to the care of the infants and made it safer.

80% of the nurses reported that it was very easy to read and send messages through the application forms or through the dialogue box. However, only 60% of the nurses checked the families’ messages daily, 20% only once a week, and one nurse even less often. Only one nurse (20%) sent messages to the families daily, 3 nurses (60%) approximately once a week, and one (20%) less often.

Although four (80%) of the nurses thought the application had the right amount of functions and one (20%) had no opinion, the results showed that very few of the functions were utilized during the study. Almost 70% of the functions were practically not used at all. Those functions which were used were choosing patients, reading and sending messages, adding patients, viewing diagrams, and registering measurements.

When asked whether they would use the web application outside of the study, 3 nurses (60%) answered yes, 2 (40%) maybe. In the questionnaires, the nurses commented the use as follows:

“It is good to have a dialogue with the parents even on the days when I do not make a home visit to them. I can get an idea of how they feel. It is good that the parents can see the weight curve. Also good to have information and advice about the premature infants easily accessible for the parents on the webpage.”

“Some parents have used the possibility of sending messages which has been useful.”

“I am not sure about the usefulness of the web form which the parents are supposed to answer every day.”

“I am struggling with my lack of motivation. I need more time to get used to the study.”

#### Nurses’ opinions about using Skype

Only two nurses used Skype during the study. One of them thought that Skype was easy to learn and use, whereas the other one thought it was neither easy nor difficult. Both nurses felt that the families had appreciated the video calls, and they expressed an interest in using Skype for communication with families also outside of the study. Both nurses considered that the sound quality was good and the image quality medium. Some technical problems related to the Internet connection at the hospital had occurred.

#### Discussions with the nurses

The study was discussed with the nurses on several occasions prior to, during, and after the study. The attitudes expressed by the nurses reflected the results of the questionnaire. Two of the nurses were clearly enthusiastic about the possibility of using ICT in home health care and also had suggestions about how to use it. Two others were moderately interested in the use of ICT and saw the study as potentially positive, although they expressed some doubts about the usefulness of ICT. One nurse was not very motivated but accepted the study as a part of her work. One of the nurses, on the other hand, had a clearly negative attitude. She felt that the use of ICT was a threat to the personal relationship between the parents and the nurse and was afraid that the use of ICT would lessen her possibility of visiting the families. She also expressed the opinion that the use of the internet and mobile phones by the families should be discouraged in general, since these activities, according to her, took families’ attention away from their infant. She was reluctant to learn the use of the web application and Skype and felt that telecommunication would increase her work load, whereas the other nurses reported that viewing the data sent by the parents and answering their questions took less than 10 minutes of work time daily and that Skype calls took approximately the same amount of time as regular telephone calls that the parents received in the control group.

## Discussion

We report here the attitudes, satisfaction, and experiences of parents of preterm infants and home health nurses about home health care supplemented with the use of a web application or Skype calls.

Although the results are based on a limited number of families, most of the answers point in the same direction. Families generally seemed very happy with the care they were given in the home health care program. The families felt confident taking care of their infant at home despite the infants’ early discharge from hospital care. This is promising considering the previous studies showing high anxiety, insecurity and stress in parents after discharge from NICU [8-10]. Combining regular home visits with the possibility of calling the nurses at any time seems to be a good method of helping families through the transition into life at home.

Even though all families were satisfied with the care at home independent of study group, the addition of ICT was appreciated in most cases. This was especially obvious in the Skype group, where all the families felt that Skype had helped them feel more confident with the care of their infant. The fact that 62.5% of the families thought the home visits could be reduced and one family even thought they could be replaced altogether by Skype indicates how useful the video conferencing was felt to be. If a larger study confirms that the use of video conferencing decreases the need of home visits, more families could be included in home health care, making it more cost-effective. Using video conferencing may also make it possible to include in the home health care service families living so far away from the hospital that home visits are impracticable. Similarly, a nonrandomized small study showed that video conferencing helped parents feel confident in the care of their premature child at home [12]. In that study families only had support through the video conferencing system, not complemented by visits by a nurse [12]. In contrast to the present study, Lindberg et al. used a dedicated video conferencing system, a more expensive and less mobile solution [12]. Two studies using video conferencing between hospital staff and families with infants with congenital heart disease showed that the use of video calls reduced the need of hospital visits for check up and reduced parental anxiety [13,14]. A study comparing the use of telemedicine for parents of infants in the NICU with a control group without access to this program also showed that telemedicine improved family satisfaction with inpatient care of very low birth weight infants [15].

Although families in the web group were positive to the use of the web application, they appeared less enthusiastic than those in the Skype group. Some of the families felt that their baby was doing so well that they did not feel motivated to answer the same questions every day, since their answers were similar on most days. Whereas some families used the application to ask the nurse questions, others felt that it was easier to call the nurse directly on the phone and get an answer right away. One problem with the web application was the fact that some families received no feedback from the nurses on the information they entered. The lack of motivation and passivity of some of the nurses was problematic. Although the nurses were supposed to read the health report and questions sent by the parents at least on the days when they were not making a home visit to the family, some nurses seldom answered the family.

The parents, who were younger and who reported more experience with computers than the nurses, also embraced the use of telecommunication in the home health care more readily than some of the nurses. The attitudes of the nurses towards the use of the web application and Skype varied from enthusiastic to very reluctant. Two nurses informed and recruited patients to the study much more actively than the others. They also reported that the families were more interested in the study and had a higher opinion of the study than most of the others. It seems evident that nurses with an enthusiastic attitude towards the study delivered information about the study in a more encouraging way which in turn affected the families’ willingness to participate. These nurses also used the web application and Skype actively. Two other nurses saw the potential of the study to improve health care while expressing some concerns, one nurse accepted the study as part of her responsibilities whereas one saw the study as a threat and avoided using the telecommunication methods. Similarly, in a maternity-care service called Net Clinic where patients and caregivers could communicate bilaterally using the Internet, three types of attitudes from the nurses were detected [22]. “Doubters” were reluctant to use NetClinic and did not believe in their own ICT competence. “Accepters” agreed that progress in ICT would inevitably affect their work. They were not necessarily enthusiastic about developing their work methods, but did what was necessary to continue their work. Development was difficult, and the nurses were not aware of the benefits of ICT and expected Net Clinic to increase their workload. “Future confidents” saw ICT as a useful tool for developing maternity care and saw it as an opportunity to participate in that development. A Dutch study using tele-monitoring in the care for chronic patients in the Netherlands similarly found that some nurses worry that implementation of telecare impoverishes patient care and hinders the development of a personal relation between nurse and patient [23]. However, studies show that telecare can lead to more frequent contact between nurses and patients [23]. Instead of impoverishing the care for patients, patients felt very close to the nurse, because there was a daily contact [23]. Training and motivating nursing staff to use ICT is a major prerequisite for the development of eHealth.

## Conclusions

This paper covers results from a study on the use of eHealth applications designed for neonatal care in the home. Results indicate that use of video conferencing was greatly appreciated by the families and was felt to reduce the need of home visits. Using a web application for daily collection of data was another potentially useful alternative. Although the nurses generally adapted well to the use of ICT, motivating some of the nurses was a challenge.

## Abbreviations

D: Day;HD: High definition;ICT: Information and communication technology;NICU: Neonatal intensive care unit;SEM: Standard error of the mean;wk: Week

## Competing interests

There are no known competing interests in this study.

## Authors’ contributions

All authors participated in the planning of the study. AG, KL, BAS, and KB designed the questionnaires. AG was in charge of the technical support, and designed the web application together with KL and BAS. AG trained the nurses in the use of ICT. AG and KB analyzed the results and were the main authors of the manuscript. HW interviewed the families. KL, BAS and KB supervised and were responsible for the project. They took part in all decisions on design and analysis, and were also the main providers of funding. EH was medically responsible for the project. All authors approved the manuscript.

## Pre-publication history

The pre-publication history for this paper can be accessed here:

http://www.biomedcentral.com/1472-6947/13/22/prepub
